# Genome-Wide Patterns of Homozygosity and Heterozygosity and Candidate Genes in Greek Insular and Mainland Native Goats

**DOI:** 10.3390/genes16010027

**Published:** 2024-12-27

**Authors:** Valentina Tsartsianidou, Antonis Otapasidis, Spiros Papakostas, Nikoleta Karaiskou, Sotiria Vouraki, Alexandros Triantafyllidis

**Affiliations:** 1 Department of Genetics, Development & Molecular Biology, School of Biology, Aristotle University of Thessaloniki, 54124 Thessaloniki, Greece; antotapas99@gmail.com (A.O.); nikolbio@bio.auth.gr (N.K.); atriant@bio.auth.gr (A.T.); 2 Genomics and Epigenomics Translational Research (GENeTres), Center for Interdisciplinary Research and Innovation (CIRI-AUTH), Balkan Center, 57001 Thessaloniki, Greece; 3 Department of Science and Technology, International Hellenic University, 57001 Thessaloniki, Greece; spapakostas@ihu.edu.gr; 4 Laboratory of Animal Husbandry, School of Veterinary Medicine, Faculty of Health Sciences, Aristotle University of Thessaloniki, 54124 Thessaloniki, Greece; svouraki@vet.auth.gr; 5 Laboratory of Animal Production, Nutrition and Biotechnology, Department of Agriculture, School of Agriculture, University of Ioannina, 47100 Arta, Greece

**Keywords:** Greek goats, runs of homozygosity, runs of heterozygosity, hotspots, candidate genes

## Abstract

Background: Runs of homozygosity (ROHs) and heterozygosity (ROHets) serve for the identification of genomic regions as candidates of selection, local adaptation, and population history. Methods: The present study aimed to comprehensively explore the ROH and ROHet patterns and hotspots in Greek native dairy goats, Eghoria and Skopelos, genotyped with the Illumina Goat SNP50 BeadChip. SNP and functional enrichment analyses were conducted to further characterize hotspots and the candidate genes located within these genomic regions. Genetic relationships between and within breeds and inbreeding coefficients were also evaluated. Results: Clear genetic differentiation and diversified management practices were depicted between the two native populations. The ROH and ROHet average genome coverage for Skopelos (65.35 and 35 Mb) and Eghoria (47.64 and 43 Mb) indicated differences in mainland and insular goats, with Skopelos showing more long ROH fragments, reflecting its geographic isolation and small population size. An ROH hotspot (CHR12: 43.59–44.61 Mb) detected in the Skopelos population has been also reported across European goats and co-localizes with a selection signal detected in the Egyptian Barki goats and sheep adapted to hot–arid conditions. A novel ROH hotspot (CHR18: 60.12–61.81 Mb), shared among the Greek breeds, harbors candidate genes enriched in biosynthesis, metabolism, and immune response. Two well-conserved ROHet islands were detected in Greek goats on chromosomes 1 and 18, with genes participating in development and embryogenesis. The Eghoria population showed the highest number of ROHet islands, potentially reflecting its adaptability to diverse environments. Conclusions: These findings offer new insights into the environmental adaptation and artificial selection in Greek goats and could be utilized in future breeding strategies for sustainable goat farming.

## 1. Introduction

The identification of continuous homozygous chromosomal segments, known as runs of homozygosity (ROHs) [1], is increasingly utilized for understanding the genome variation and evolutionary history of livestock populations. An ROH is present in individuals due to the inheritance of identical by descent (IBD) haplotypes from a common ancestor [2]. These consecutive segments have been specifically used to explore inbreeding events, demographic processes, and the effects of natural and artificial selection [3,4,5], shaping the genome structure of livestock populations. The assessment of ROH distribution, frequency, and length across the genome enables the detection of recent (long ROH) and ancient inbreeding (short ROH) events. ROH hotspots, or ROH islands [6], have been identified in regions associated with domestication, selection, and local environmental adaptation in various livestock species, including goats [7,8,9,10], cattle [11,12,13,14,15,16], sheep [17,18,19], pigs [5], and poultry [20]. The estimation of individual inbreeding levels based on an ROH is also an accurate approach for implementing mating practices to prevent inbreeding depression in livestock populations [21]. Recently, the phenomenon of purging, which is the interaction between selection and inbreeding to at least partially eliminate the inbreeding load of a population, has been reviewed for its potential favorable impact on animal fitness and inbreeding depression events. However, its application should be treated with caution, especially for domesticated animals [22].

Runs of heterozygosity (ROHets), also referred to as heterozygosity-rich regions (HRRs), are genomic regions with increased clustered heterozygosity. These chromosomal regions have been associated with important functional traits, such as immune response, disease resistance, survival, and overall animal fitness [4,6,17]. Patterns of clustered heterozygosity have been explored in cattle [4,23,24,25], sheep [6,17], goats [26,27,28], pigs [29,30], and horses [31]. However, these segments are less studied compared to ROHs in livestock populations.

Despite the significance of ROH and ROHet studies, these genomic features have never been thoroughly explored in Greek native goats. Greece has a long tradition of goat farming, dating back ~6500 years [32], and currently holds the largest goat population in the European Union, with 2.87 million goats [33]. Most of these goats are reared in low-input semi-extensive farming conditions in mountainous or semi-mountainous areas with variable environmental conditions [34]. The Greek national herd is represented by two dairy goat breeds, Eghoria and Skopelos, along with a few foreign breeds and their crosses. The Eghoria breed consists of 600,000 animals [35] characterized by large phenotypic variability and adaptability across diverse environmental conditions within Greece. The Skopelos breed, an insular homogeneous population mostly located in the Northern Sporades Island complex, comprises 10–11 thousand animals [36] with distinctive brown coat color and high adaptability to environmental changes [37].

In recent years, the high-yielding cosmopolitan breeds (e.g., Saanen, Alpine, Toggenburg, and Anglo-Nubian) that have been introduced in Greece [34] are used in uncontrolled crossbreeding with indigenous goats. This practice can lead to a decline in the population size of purebred native individuals, loss of genetic diversity, and potential loss of unique genetic traits associated with economic importance and local adaptation. Given the vulnerability of the Mediterranean region to climate changes, including increased summer temperatures and dry weather events [36], exploring and protecting locally adapted populations is crucial for the anticipation of such changes and sustainable livestock farming [38].

Therefore, this study aimed to investigate the genome diversity in Greek Eghoria and Skopelos goat breeds, exploring the ROH and ROHet patterns. We sought to identify the ROH/ROHet number, distribution, length, and hotspots potentially involved in biological processes related to local adaptation and selection. Additionally, we assessed genome-wide inbreeding and genetic relationships between the studied Greek goats to provide general and comparative insights into the observed ROH/ROHet patterns, contributing to future conservation management actions.

## 2. Materials and Methods

### 2.1. Goat Samples, SNP Genotyping, and Data Editing

**Figure 1:**
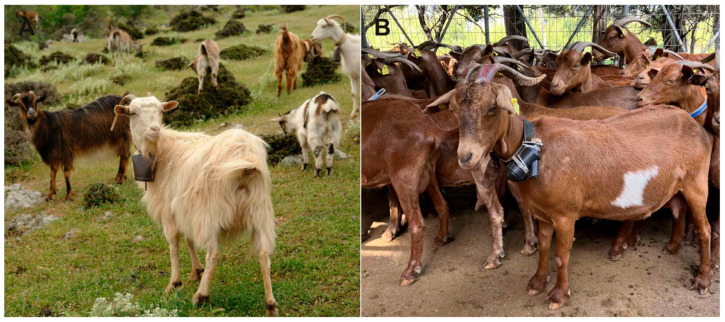
Representative animals of the Greek goat breeds sampled within this study. (**A**) Greek Eghoria goat and (**B**) Skopelos goat.

A total of 70 Eghoria (Figure 1A) and 289 Skopelos (Figure 1B) goats from 4 farms, located in Northern mainland and insular Greece, as illustrated in Figure S1, were studied. Blood samples were collected from the jugular vein in EDTA vacutainers. DNA was extracted using GeneJet Whole Blood Genomic DNA Purification Mini Kit (Thermo Scientific, Waltham, MA, USA). All DNA samples were genotyped with the Illumina Goat SNP50 BeadChip featuring 53,327 single nucleotide polymorphisms (SNPs). SNP data, available at SMARTER-database [39], were filtered using PLINK v1.9 software [40]. Marker and sample call rate thresholds were applied at 98% and 95%, respectively. SNP markers localized on sex and zero chromosomes were removed. To detect runs of homozygosity and heterozygosity at the within-breed level, SNPs were not filtered for low MAF, as proposed for medium-density SNP data [41], resulting in 47,322 available SNPs. SNP markers with minor allele frequency of less than 2% and samples with genomic relatedness up to second degree were removed using KING software v2.3.1 (--king-cutoff 0.0884) [42] to estimate diversity metrics and inbreeding coefficients with a final dataset of 189 animals and 46,806 SNPs. SNP positions were mapped according to the ARS1.2 genome assembly.

### 2.2. Genome-Wide Diversity and Principal Component Analysis

The expected (He) and observed heterozygosity (Ho) were measured using PLINK 1.9 software [40]. The inbreeding coefficient (*F*_IS_), based on the difference between the observed and expected number of homozygous genotypes under the Hardy–Weinberg equilibrium, was also estimated for the Greek populations to compare with ROH-based results. To obtain a general overview of the genetic relatedness between and within the studied Greek goat populations and samples collected, principal component analysis (PCA) was also conducted. The GEMMA 0.98.1 software [43] was used for this purpose, and the decomposed genetic relatedness matrix (GRM) was then illustrated.

### 2.3. Detection of Runs of Homozygosity and Heterozygosity

ROH and ROHet regions were detected using the R package detectRUNS v0.9.5 [4]. The sliding window (SW) approach was applied for the ROH identification, and the consecutive-runs method (CR) for ROHet detection, respectively. The SW method, which relies on defined SNP windows scanning the genome, is preferable for identifying homozygous segments. The CR method, evaluating each SNP locus individually, is more suitable for ROHet detection [4,16,31]. ROHs are typically longer, continuous segments, whereas small or usually fragmented heterozygosity-enriched regions, often ‘interrupted’ by a few homozygous SNP loci, may be missed or have their boundaries underestimated by SW.

The parameters used to identify ROHs were set in concordance with previous studies that explored ROH patterns in goats [3,44]: (i) a sliding window size of 15 SNPs; (ii) a minimum number of 15 consecutive SNPs per run; (iii) a minimum ROH length of 500 kb; (iv) a maximum gap of 250 kb between two consecutive homozygous SNPs; (v) a minimum density of 100 kb; and (vi) one heterozygous genotype and 1 missing SNP allowed within an ROH. The default settings were followed for all other parameters.

The genomic inbreeding coefficient based on ROH (*F_ROH_*) was estimated for each individual, as described by McQuillan et al., (2008) [45]:

(1) $$F_{ROH}\;=\;\frac{{\sum L}_{ROH}}{L_{aut}}$$

where ∑*L_ROH_* is the sum of the length of all ROHs detected in an individual, and *L_aut_* is the total length of autosomal genome according to the DNA array used for genotyping.

The parameters used to identify ROHet segments were set in concordance with a recent study exploring heterozygosity-rich regions in commercial and local goat breeds [46]: (i) a minimum number of 15 consecutive SNPs; (ii) 1 Mb maximum gap between two consecutive SNPs; (iv) a minimum length of heterozygous regions 250 kb; and (v) 3 homozygous SNPs and 2 missing SNPs allowed within an ROHet.

Highly homozygous and heterozygous regions were designated as ROH/ROHet islands by setting the top 1% of SNP frequency of occurrence within the ROH/ROHet regions across the studied individuals [47] using the tableRUNS() function. The frequency thresholds were 0.1514 and 0.1831 for ROH and ROHet islands, respectively.

### 2.4. Gene and Functional Annotation

The extent of linkage disequilibrium was determined in regions where ROH and ROHet hotspots were identified. Pairwise LD estimation (r^2^) was carried out using PLINK 1.9 software [40]. SNP annotation was then performed using the Variant Effect Predictor (VEP) [48] tool (Ensemble release 108) using an annotation window of 250 kb upstream and downstream from the SNPs, based on the average pairwise LD and average distance between the SNPs detected in the ROH and ROHet islands. Functional annotation analysis was performed for all identified genes using KEGG, ENSEMBL’s gene library [49], and DAVID bioinformatics resource [50]. Subsequently, functional enrichment analysis was conducted using the gProfiler genomic tool [51] to identify candidate annotated genes involved in biological processes. Terms with FDR-adjusted *p*-values < 0.05 were provided by gProfiler tool, using Benjamini–Hochberg method for multiple testing correction.

## 3. Results

### 3.1. Genome-Wide Diversity and Principal Component Analysis

The average expected and observed heterozygosity estimates measured for the Eghoria (Ho = 0.421, He = 0.422) and Skopelos (Ho = 0.389, He = 0.396) unrelated goats (a subset of 189 samples) indicate similar levels of polymorphism to other Mediterranean populations. Additionally, heterozygosity levels of the Skopelos insular breed were lower compared to the Greek Eghoria animals raised in Northern mainland Greece.

**Figure 2:**
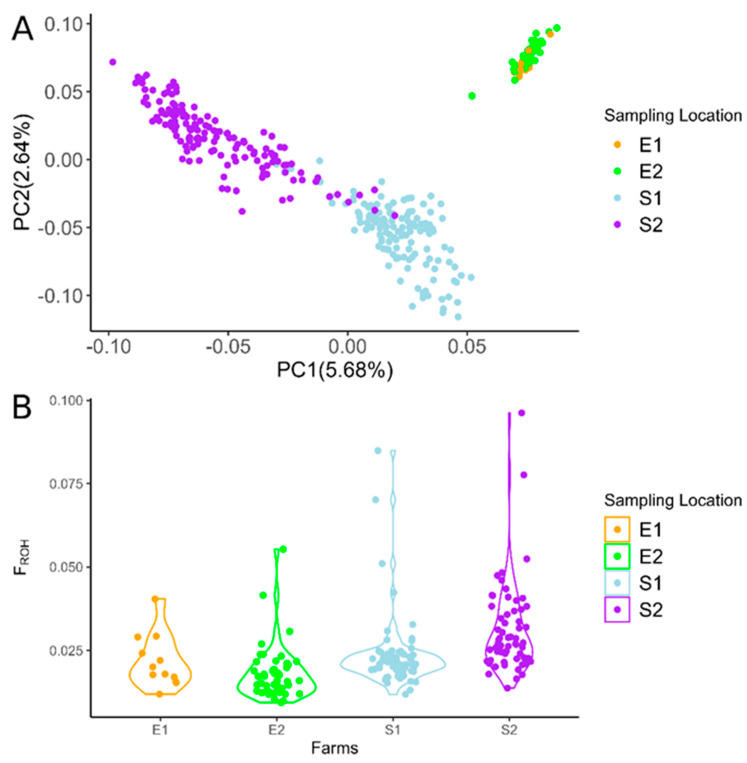
(**A**) PCA plot of Greek goats colored by farm of origin. E1, E2: Eghoria, S1, S2: Skopelos. (**B**) Individual run of homozygosity (ROH)-based inbreeding levels (*F_ROH_*) in Greek goats. E1, E2 farms correspond to the Eghoria breed, and S1, S2 farms correspond to the Skopelos breed.

The genetic structure of Greek populations was investigated at a local scale using the total number of genotyped goats, where the two breeds were well differentiated from each other (Figure 2A). The first two principal components collectively explained 8.32% of the total variance (PC1 = 5.68%, PC2 = 2.64%). Skopelos individuals were represented by two scattered clusters attributed to the insular sampling areas of Alonnisos and Skopelos (farms S1, S2). A small subset of animals showed a genetic structure identical to that of individuals raised on the other island. On the contrary, the Eghoria goats, sampled from two mainland locations in Northern Greece (farms E1, E2), were very closely clustered with a strong population stratification at the within-breed level. The individuals phenotypically mislabeled as “Eghoria” and identified as “Skopelos” through PCA were excluded from the ROH and ROHet analyses.

### 3.2. ROH and ROHet Patterns

The average ROH-based inbreeding coefficient (*F_ROH_*), based on the total observed ROHs, was estimated identical in Eghoria (*F_ROH_* = 0.019, SE = 0.001) and Skopelos (*F_ROH_* = 0.027, SE = 0.001) breeds. Slight differences were estimated with the average Wright’s inbreeding coefficient between Eghoria (*F*_IS_ = 0.003, SE = 0.003) and Skopelos goats (*F*_IS_ = 0.017, SE = 0.001). The inbreeding levels based on the ROH per individual were also illustrated according to the farm of origin, indicating more inbred animals raised in farms S1 and S2 of the Skopelos breed compared to farms E1 and E2 of Eghoria goats (Figure 2B).

**Figure 3:**
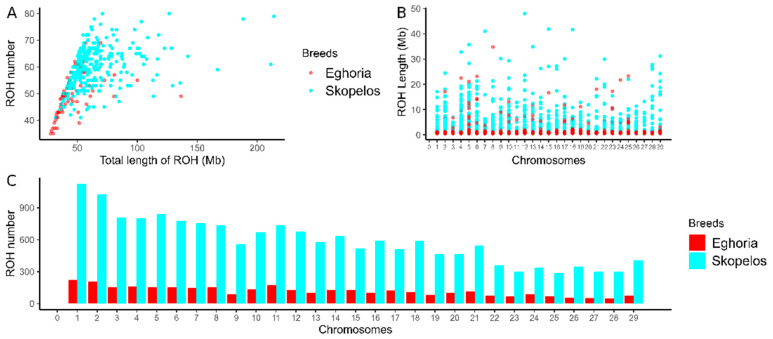
ROH distribution in Greek goat breeds. (**A**) Total ROH number per animal and breed compared to the total ROH length. (**B**) Average ROH length in each autosomal chromosome. (**C**) Total ROH number per chromosome. Color attributes to the breed of origin.

A total of 20,307 ROHs were identified in the genome of the 351 studied native goats. Of these, 17,020 were found in Skopelos and 3287 in the Eghoria breed with an average of 56 and 46 ROHs per animal, respectively. Additionally, the average ROH length was 1.09 Mb in Skopelos and 948.17 kb in the Eghoria population. Figure 3A shows the relationship between the total number of ROHs and the genome coverage covered by the ROHs in each individual, indicating high variability within both breeds, with higher average genome coverage in Skopelos (63.16 Mb) compared to Eghoria goats (45.26 Mb). The highest individual genomic coverage was estimated at 213.94 Mb in Skopelos and 136.50 Mb in the Eghoria breed. Figure 3B,C illustrates the ROH genome coverage and ROH number in each chromosome for each breed. The longest length of single ROH segments was identified on chromosomes 12 (48 Mb) and 8 (34.8 Mb) in Skopelos and Eghoria, respectively (Figure 3B). The ROH number per chromosome decreases in consistency with chromosome length, with exceptions in both breeds. This trend is more evident in the Skopelos breed (Figure 3C).

**Figure 4:**
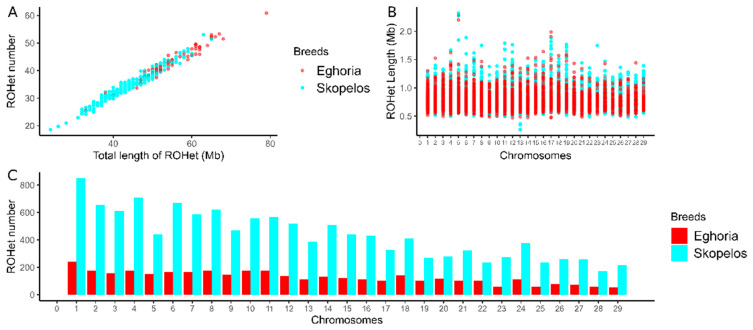
ROHet distribution in Greek goat breeds. (**A**) Total ROHet number per animal and breed compared to the total ROH length. (**B**) ROHet length in each autosomal chromosome. (**C**) Total ROHet number per chromosome. Color attributes to the breed of origin.

A total of 16,269 ROHets were identified in the studied sample of animals, with 12,574 segments in Skopelos and 3695 in the Eghoria population. On average, the ROHet number per animal identified at the within-breed level was 44 and 55 for Skopelos and Eghoria. A similar average ROHet length was estimated for Skopelos (786.14 kb) and Eghoria goats (785.93 kb). Figure 4A illustrates the total number of ROHets compared to the genome coverage covered by the ROHets in each individual, as previously depicted for ROHs. The average ROHet genomic coverage was higher in Eghoria (43.34 Mb) compared to the Skopelos population (34.99 Mb) (Figure 4A), with an average per individual ranging between 40 to 50 Mb in the native breeds. Figure 4B,C illustrates the ROHet genomic coverage and the ROHet number in each chromosome for the two studied breeds. The longest heterozygous segments were identified on chromosome 5 (SKO: 2.32 Mb, EGH: 2.29 Mb), whereas the shortest were spotted on chromosome 13 for Skopelos (508.67 kb) and chromosome 8 for Eghoria (508.76 kb) (Figure 4B). Chromosome 1 presented the highest number of ROHets for both breeds (Figure 4C).

ROH/ROHet distribution by size class (ROH/ROHet length) is also shown in Table 1 for both breeds. The majority of ROHs found ranged between 0.5 to 6 Mb, indicating an inverse trend between ROH number and length. Most of the ROHets were classified in the size class 0.4–0.8 Mb. The defined size classes were selected to highlight the presence of long ROH segments (12–24 Mb, 24–48 Mb), especially in the insular Skopelos goat breed. ROHet size classes were determined considering the shorter length of heterozygous segments to ensure a better representation of our findings.

**Figure 5:**
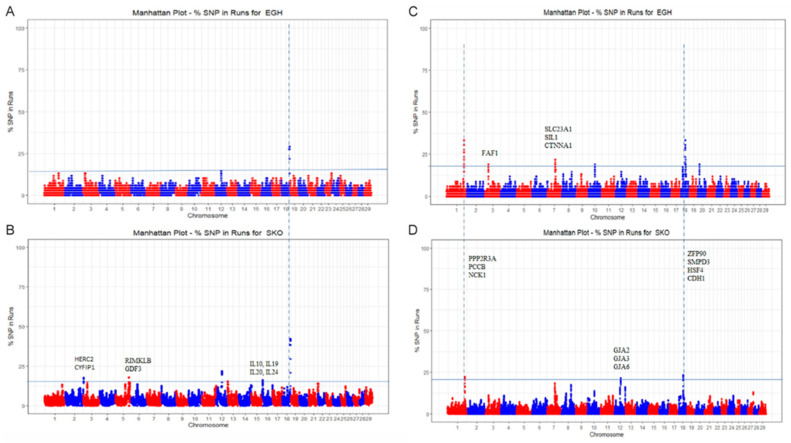
Manhattan plots depicting the SNP frequency distribution within an ROH and ROHet region in the Eghoria (**A**,**C**) and in Skopelos goats (**B**,**D**), respectively. Blue horizontal lines indicate the top 1% frequency threshold, corresponding to 15.141 for ROH and 18.309 for ROHet. Vertical dashed lines highlight shared islands across Greek breeds. Representative candidate genes are also indicated.

Figure 5 shows the frequency of SNPs within an ROH or an ROHet across the autosomal chromosomes for both Greek goat breeds, revealing highly homozygous and heterozygous genomic regions. These regions, defined as ROH and ROHet islands, were detected by extracting the top 1% of the SNP frequency distribution within the respective segment. Six ROH islands (EGH:1, SKO:5) and nine ROHet regions (EGH:6, SKO:3) were identified (Table 2). An ROH island of 1.66 Mb was found on chromosome 18 in the Eghoria breed, including 19 SNPs and 33 candidate genes. In the Skopelos breed, such genomic regions were detected ranging from 161.26 kb to 1.69 Mb and including 4–21 SNP markers, where 106 candidate genes were localized (Table S1). Notably, the two native breeds share an ROH island identified on chromosome 18 with 19 common SNP markers. Six ROHet regions were identified in Eghoria, ranging in length from 121 kb to 1 Mb, including 7–18 SNPs and harboring 103 candidate genes. Three heterozygosity-rich regions were identified in Skopelos, with shorter lengths compared to the Eghoria population (531.44–746.94 kb), harboring 5–12 SNP markers and 183 candidate genes (Table S2). Moderate to low pairwise LD (r^2^) values were estimated for the SNP markers lying within the ROH (r^2^ = 0.24–0.52) and ROHet regions (r^2^ = 0.27–0.4) in both breeds (Table 2).

### 3.3. Gene Identification and Functional Annotation

SNP annotation revealed a total of 299 candidate genes within or upstream/downstream (±250 kb) of the identified ROH (*n* = 106) and ROHet (*n* = 193) regions in both indigenous Greek goat breeds. Representative genes are presented in Figure 5. Candidate genes detected within the ROH islands were significantly enriched (*p* < 0.05) in 101 GO terms (EGH: 39, SKO: 62) (Table S3). Within all ROH islands, 23 genes have been identified to participate in regulatory, metabolic, biosynthetic, and immune response pathways in both breeds. Additionally, genes found in the Skopelos genome’s ROH islands are involved in the regulation of immune response (*IL10*, *IL19*, *IL20*, and *IL24*) and negative regulation of heterochromatin formation/regulation (*APOBEC1*, *AICDA*). Importantly, a set of 11 genes encoding for zinc finger proteins and involved in the HSV-1 infection pathway is located within the ROH island (1.6 Mb) identified on chromosome 18 in both populations. This finding implies that the identified genomic region may participate in the innate immune response of goats. Furthermore, genes localized on the ROH islands of chromosomes 2 and 16 have previously been associated with innate and acquired immune responses, cytokine cycling in sheep (*HERC2*, *CYFIP1*) [52], and heat response (*IL10*) [53].

A total of 57 GO terms with an FDR-adjusted *p*-value < 0.05 were identified for the candidate genes closely located at the ROHet regions (EGH: 16, SKO: 41). The indicative GO terms are described in Table S4. The candidate genes were enriched in diverse biological processes, such as phosphatase (*NCK1*) and binding (*NCK1*, *CTCF*, *THAP11*, *NFATC3*, and *ZFP90*) activity, growth development (*FAF1*) [54], metallopeptidase activity (*DPEP2* and *DPEP3*), transport activity and ear development (*GJB2* and *GJB6*), physical development (*GJA3*) [55], and metabolism (*HSD11B2*). Indicatively, several genes have been reported in previous studies to participate in reproduction (*STAG1* and *PCCB*) [56], heat response (*HSF4*) [57], skeletal and embryonic viability and development [3,54], regulation of neurogenesis, and Seckel syndrome *(CENPJ*) [58,59].

## 4. Discussion

Runs of homozygosity (ROHs) have been extensively explored since homozygosity has been linked to individual demography and the genetic architecture of animal traits [1]. Despite the growing interest in heterozygous segments (ROHets) and their relevance to population demographics and adaptive history, they are still understudied in animal genetic resources compared to ROHs. It is also important to note that both ROH and ROHet distribution, length, and frequency have not been investigated in livestock populations reared and locally adapted in Greece, except for the highly producing Greek Chios sheep [17]. This study aimed to assess the genome variation and to identify regions potentially associated with local adaptation and directional selection in the Greek native goats, exploring the homozygosity (ROH) and heterozygosity (ROHet) patterns across their genome for the first time. The inter- and intra-population genetic relationships, inbreeding coefficients, and genetic diversity metrics were also assessed to provide a general overview of these native populations.

Both of the Greek goat populations showed similar levels of genetic diversity with previous findings [59]. The estimated heterozygosity exceeded the global (Ho = 0.369, He = 0.378) and European goat diversity average (Ho = 0.355, He = 0.379) [60]. Heterozygosity levels were close to those observed in neighboring Mediterranean [60], Egyptian [60], Turkish [3], and southern African breeds [61]. These results were in concordance with low average inbreeding coefficients (*F*_IS_ = 0.003–0.017, *F_ROH_* = 0.019–0.027) estimated for the Greek breeds. Specifically, ROH-based inbreeding levels were comparable to Mediterranean, West Asian, and North African breeds, ranging from 0.02 to 0.03 [3]. Inbreeding and heterozygosity levels indicated that the Skopelos population, mainly located and sampled from insular northern Greece, is more inbred compared to the Eghoria breed, potentially attributed to its geographic isolation and limited gene flow. The genetic distances observed between the two Greek populations, reflecting their sampling positions from northern Greece (Figure S1), verified the genetic differentiation between the two dairy goat populations. Skopelos individuals also showed evident differentiation based on their farm of origin, an expected outcome since these animals were collected from distinct islands. On the other hand, Eghoria goats sampled from two adjacent farms were very closely clustered, indicating animal exchange and gene flow. These results suggest different management practices for the insular and mainland studied goat breeds. An extensive representative sampling across Greece could shed more light on the genome diversity of the Greek Eghoria breed and the potential subpopulations existing due to geographic isolation and adaptation to variable environmental conditions.

The majority of ROH segments were short (0.5–6 Mb), indicating more ancient inbreeding events [62]. The Skopelos breed demonstrated a higher number of short and long ROHs, resulting in higher average ROH genome coverage (63.16 Mb) compared to the Greek Eghoria population (45.26 Mb) (Figure 3B). The ROH patterns highlight the impact of geographic isolation on the genomic makeup of insular populations, as previously described [3]. It is noteworthy that these results are more representative of the Skopelos breed, given the number of animals genotyped in comparison to Eghoria goats. The average ROH number per individual for native Greek goats (*n* = 51) is close to the European mean (*n* = 56) and the South European populations (*n* = 49) [63] and lower than the northern European populations [3]. On the other hand, the average ROH genome coverage (~54 Mb) is lower than the European (277 Mb) and the North European average (183 Mb) [3,63], indicating lower inbreeding levels in Greek goats.

Six highly frequent ROH segments were identified (SKO:5, EGH:1) in Greek goats. The ROH hotspot detected on chromosome 12 (43.59–44.61 Mb) has also been reported as abundant across goat breeds originating from central and south Europe (43.63–44.53 Mb) [63]. This region also overlaps with the putative selection signature detected in the Egyptian Barki goats adapted to hot-arid environmental conditions [64]. The ROH hotspot detected on chromosome 18 (60.12–61.81 Mb) in both Greek goat breeds has not been previously reported. Several genes are localized and enriched in biosynthetic, regulatory, metabolic, and immune system processes. Specifically, we identified a set of 11 novel genes encoding for zinc finger proteins localized within the ROH island on chromosome 18 in both Greek breeds associated with an adaptive response of the innate immune system to HSV-1 virus infection, based on the KEGG Pathways’ database. The livestock populations in developing countries, including Greece, face various endemic diseases and disease outbreaks since dense livestock populations favor the transmission of microbes across individuals. The presence of many immune system-related genes in this particular ROH island could reflect selection for disease resistance to potential endemic diseases that may arise [65]. Additionally, the immune system-related *IL10* gene, located within the ROH island identified on chromosome 16, has previously been associated with heat response in small ruminants [53,56], while the *RIMKLB* gene on chromosome 5 has been linked to metabolic pathways in Skopelos goats. Both cases indicate a potential association with the environmental adaptation of the insular Skopelos populations to semi-arid less favored conditions.

Herein, we identified 16,269 ROHets of variable lengths (0.4–3.2 Mb) across the genome of the native populations. The average number of detected ROHets per goat in Eghoria (*n* = 55) and Skopelos (*n* = 44) breeds were similar to that of locally adapted goat populations studied before, like Barki, Creole, and Landrace (*n* = 51), in contrast to the higher length detected in commercial goats (*n* = 83.3) [46]. Furthermore, a similar average ROHet length was estimated between Greek populations (786.54 kb) and the above-mentioned local breeds (794.5 kb). Within our study, nine ROHet hotspots were identified across the genome of Greek goats (EGH: 6, SKO:3). The average genomic coverage was higher in Eghoria (43.34 Mb) compared to the Skopelos population (34.99 Mb). The presence of several highly heterozygous regions in the Eghoria breed underscores the diverse genetic profile of this population, characterized by extensive phenotypic and adaptive variation spread across Greece. In contrast, fewer ROHet islands were detected for the insular Skopelos goats, indicating the impact of geographic isolation. These results are consistent with the diversity and inbreeding estimates reported for both studied breeds. Many of the identified ROHets overlap with previously studied heterozygous segments linked to survival and development [26]. The presence of favored heterozygosity shared across global goat breeds and clustered in specific chromosomes indicates genomic regions under balancing selection [28,46]. In detail, two overlapping heterozygosity-rich regions were observed between Greek populations on chromosomes 1 (131.93–132.50 Mb) and 18 (36.28–37.01 Mb) that both align with highly heterozygous regions identified in multiple goat breeds [26,28,63]. Remarkably, the ROHet hotspot located on chromosome 1 has been detected across global goat breeds, associated with embryonic development (*STAG1*, *PCCB*) and heterotic balancing selection, suggesting an important role in goat domestication [26,28]. The ROHet hotspot detected on chromosome 18 has recently been reported as highly heterozygous in Italian and Alpine breeds [28] and highly homozygous in Asian, European, and African goats [63], respectively. It has also been suggested under selection for fiber production in goats [7]. This contrasting genetic variation suggests different selective pressures across this region according to the selected breeding strategies and diversified production purposes in goat species [26]. The candidate genes within this ROHet on chromosome 18 were significantly enriched in biological processes related to DNA-binding, metallopeptidase, and lipase activity. Furthermore, the ROHet region identified on chromosome 12 (49.88–50.63 Mb) was present only in the Skopelos population and overlaps with a prevalent heterozygosity hotspot (49.9–51.6 Mb) shared by over 35% of goats across five improved and traditional goat breeds [8] and by Italian Alpine goats (49.80–51.28 Mb) [28]. Previous studies have also reported the same region as a hotspot for homozygosity in goat breeds [62]. Notably, this hotspot is closely located in a chromosomal region under positive selection in Boer goats [66], Egyptian Barki goats, and sheep [64]. A set of 13 annotated genes were identified within this region, including *ATP12A*, *GJA3*, *GJB2*, and *GJB6* genes, encoding gap junction proteins that affect body size, skeletal development, and embryogenesis [66]. Additionally, *GJB2* and *GJB6* participate in the nervous system, hearing functions [1,67], and ectodermal processes [66,68]. This conserved genomic region may have undergone positive selection in both sheep and goats before goat domestication potentially related to sensory perception serving feed and threat detection in the field [26]. Functional annotation of heterozygous hotspots on chromosomes 3 (24.91–25.10 Mb) and 18 (39.64–39.93 Mb), specifically identified in the Greek Eghoria breed, revealed genes involved in biological processes related to animal survival and fitness, such as bone formation and skeletal development [54], heat resistance (*HSF4*) [69], and hair follicle morphogenesis (*CDH1*) [70]. These regions co-localize with heterozygous segments also detected in European goat populations [52]. It is noteworthy that some of the identified ROHet regions, shared between Greek and global goat breeds, may indicate genomic regions with beneficial heterozygosity during goat domestication. On the other hand, regions found exclusively in the Eghoria or Skopelos population might suggest post-domestication demographic events associated with adaptation to diversified environmental demands and/or management practices.

Finally, two ROHet hotspots found in the Eghoria breed on chromosomes 7 and 20 have not been previously documented in the literature to our knowledge. Genes involved in heat stress response (*DNAJC18*) and blood pressure (*ADAMTS6*) are localized within these regions, implying their role in coping with demanding environmental conditions. However, the ROH/ROHet identification is largely dependent on bias related to SNP arrays, defined parameters of the analysis, and sample size. Our findings related to the Greek Eghoria breed may be susceptible to ascertainment bias related to the design of the Goat SNP50 BeadChip, given that only Skopelos individuals were included in the validation step. Future analyses with whole-genome data would contribute to comprehensively studying the identified genomic regions.

## 5. Conclusions

Our study explored the ROH/ROHet patterns and islands shaped within indigenous goat breeds in Greece. Low inbreeding levels were observed for Greek goats. The Eghoria breed was less inbred compared to the insular Skopelos breed, with multiple heterozygosity-rich hotspots, signifying the impact of geographical isolation, diverse environmental pressures, and mating practices on the genomic makeup of these populations. The identification of common homozygous and heterozygous hotspots between the Greek breeds, in consistency with previous findings and overlapping with selection signatures, highlights the presence of increased homozygosity and heterozygosity in specific chromosomal areas of the goat genome. Overall, these results expand our knowledge of genome variation within Greek goats and provide valuable insights to facilitate the implementation of breeding strategies that jointly protect local genetic resources and improve desirable traits.

## Figures and Tables

**Table 1:** Distribution of runs of homozygosity and heterozygosity identified in the Eghoria and Skopelos goats represented by size classes based on ROH length.

Run	Size Class (Mb)	Total Number/Total Length (Mb)	
		Eghoria	Skopelos
ROH	0–6	3363/2665.53	16,734/15,031.79
	6–12	24/181.33	183/1532.23
	12–24	15/234.97	81/1303.51
	24–48	1/34.79	22/691.21
ROHet	0.2–0.4	0/0	4/1.23
	0.4–0.8	2309/1600.44	7770/5381.66
	0.8–1.6	1380/1291.94	4839/4502.14
	1.6–3.2	6/11.65	31/54.95

Abbreviations: ROH = runs of homozygosity; ROHet = runs of heterozygosity.

**Table 2:** Description of ROH and ROHet islands identified in indigenous Greek goats. Chromosomal positions were assigned according to the ARS1.2 genome assembly.

Breed	Chr	Start (bp)	End (bp)	Length (bp)	SNPs	ALD	Genes
ROH							
EGH	18 ^1^	60,120,713	61,776,542	1,655,829	19	0.52	33
SKO	2	135,434,648	136,405,967	1,655,829	17	0.24	11
	5	99,979,645	100,140,908	161,263	4	0.24	16
	12	43,598,144	44,611,581	1,013,437	19	0.28	1
	16	4,091,765	4,282,942	191,177	5	0.21	12
	18 ^1^	60,120,713	61,813,628	1,692,915	21	0.39	33
ROHet							
EGH	1 ^2^	131,931,194	132,736,269	805,075	17	0.28	9
	3	24,908,534	25,076,519	167,985	5	0.26	3
	7	60,015,530	60,137,051	121,521	5	0.33	16
	18 ^2^	36,283,603	37,295,931	1,012,328	18	0.27	60
	18	39,636,695	39,926,799	290,104	7	0.35	4
	20	13,847,556	14,250,543	402,987	9	0.29	11
SKO	1 ^2^	131,970,293	132,501,737	531,444	11	0.39	9
	12	49,884,737	50,631,677	746,940	15	0.30	25
	18 ^2^	36,360,921	37,006,578	645,657	12	0.30	46

Abbreviations: Chr = chromosome; bp = base pairs; SNP = single nucleotide polymorphism; ALD = Average Linkage Disequilibrium; EGH = Eghoria; SKO = Skopelos; ROH = runs of homozygosity; ROHet = runs of heterozygosity. ^1^ Common ROH hotspots shared among Greek goat breeds. ^2^ Common ROHet hotspots shared among Greek goat breeds.

## Data Availability

The original data presented in this study are openly available at https://github.com/cnr-ibba/SMARTER-database (accessed on 15 May 2023).

## Supplementary Materials

The following supporting information can be downloaded at www.mdpi.com/article/10.3390/genes16010027/s1, Figure S1. Geographical map indicating sampling locations of Greek goat breeds in the present study. Farms E1 (*n* = 15) in Serres and E2 in Thessaloniki (*n* = 55) correspond to the Eghoria breed, while Skopelos goats were sampled from farms S1 (*n* = 142), S2 (*n* = 147) located in Alonnisos and Skopelos islands. Table S1. List of candidate genes detected in ROH islands (the top 0.99 percentile of at least 4 consecutive SNPs) in Greek Eghoria and Skopelos goats. Table S2. List of candidate genes detected within ROHet islands (the top 0.99 percentile of at least 4 consecutive SNPs) in Greek Eghoria and Skopelos goats. Table S3. GO terms and candidate genes detected within ROH islands in Greek Eghoria and Skopelos goats. Table S4. GO terms and candidate genes detected within ROHet islands in Greek Eghoria and Skopelos goats.

## References

[1] Ceballos F.C., Joshi P.K., Clark D.W., Ramsay M., Wilson J.F. (2018). Runs of Homozygosity: Windows into Population History and Trait Architecture. Nat. Rev. Genet..

[2] Broman K.W., Weber J.L. (1999). Long homozygous chromosomal segments in reference families from the centre d’étude du Polymorphisme Humain. Am. J. Hum. Genet..

[3] Cardoso T.F., Amills M., Bertolini F., Rothschild M., Marras G., Boink G., Jordana J., Capote J., Carolan S., Hallsson J.H. (2018). Patterns of homozygosity in insular and continental goat breeds. Genet. Sel. Evol..

[4] Biscarini F., Mastrangelo S., Catillo G., Senczuk G., Ciampolini R. (2020). Insights into genetic diversity, runs of homozygosity and heterozygosity-rich regions in Maremmana semi-feral cattle using pedigree and genomic data. Animals.

[5] Xu Z., Mei S., Zhou J., Zhang Y., Qiao M., Sun H., Li Z., Li L., Dong B., Oyelami F.O. (2021). Genome-wide assessment of runs of homozygosity and estimates of genomic inbreeding in a Chinese composite pig breed. Front. Genet..

[6] Selli A., Ventura R.V., Fonseca P.A.S., Buzanskas M.E., Andrietta L.T., Balieiro J.C.C., Brito L.F. (2021). Detection and visualization of heterozygosity-rich regions and runs of homozygosity in worldwide sheep populations. Animals.

[7] Bertolini F., Cardoso T.F., Marras G., Nicolazzi E.L., Rothschild M.F., Amills M. (2018). Genome-wide patterns of homozygosity provide clues about the population history and adaptation of goats. Genet. Sel. Evol..

[8] Signer-Hasler H., Henkel J., Bangerter E., Bulut Z., Drögemüller C., Leeb T., Flury C., The VarGoats Consortium (2022). Runs of Homozygosity in Swiss Goats Reveal Genetic Changes Associated with Domestication and Modern Selection. Genet. Sel. Evol..

[9] Manunza A., Diaz J.R., Sayre B.L., Cozzi P., Bobbo T., Deniskova T., Dotsev A., Zinovieva N., Stella A. (2023). Discovering Novel Clues of Natural Selection on Four Worldwide Goat Breeds. Sci. Rep..

[10] Zhao Q., Huang C., Chen Q., Su Y., Zhang Y., Wang R., Su R., Xu H., Liu S., Ma Y. (2024). Genomic Inbreeding and Runs of Homozygosity Analysis of Cashmere Goat. Animals.

[11] Dixit S.P., Singh S., Ganguly I., Bhatia A.K., Sharma A., Kumar N.A., Dang A.K., Jayakumar S. (2020). Genome-wide runs of homozygosity revealed selection signatures in Bos indicus. Front. Genet..

[12] Zavarez L.B., Utsunomiya Y.T., Carmo A.S., Neves H.H., Carvalheiro R., Ferenčaković M., Pérez O’Brien A.M., Curik I., Cole J.B., Van Tassell C.P. (2015). Assessment of Autozygosity in Nellore Cows (Bos indicus) through High-Density SNP Genotypes. Front. Genet..

[13] Zhang Q., Guldbrandtsen B., Bosse M., Lund M.S., Sahana G. (2015). Runs of Homozygosity and Distribution of Functional Variants in the Cattle Genome. BMC Genom..

[14] Szmatoła T., Gurgul A., Jasielczuk I., Ząbek T., Ropka-Molik K., Litwińczuk Z., Bugno-Poniewierska M. (2019). A Comprehensive Analysis of Runs of Homozygosity of Eleven Cattle Breeds Representing Different Production Types. Animals.

[15] Ghoreishifar S.M., Moradi-Shahrbabak H., Fallahi M.H., Jalil Sarghale A., Moradi-Shahrbabak M., Abdollahi-Arpanahi R., Khansefid M. (2020). Correction to: Genomic Measures of Inbreeding Coefficients and Genome-Wide Scan for Runs of Homozygosity Islands in Iranian River Buffalo, Bubalus bubalis. BMC Genet..

[16] Mulim H.A., Brito L.F., Pinto L.F., Ferraz J.B., Grigoletto L., Silva M.R., Pedrosa V.B. (2021). Characterization of Runs of Homozygosity, Heterozygosity-Enriched Regions, and Population Structure in Cattle Populations Selected for Different Breeding Goals. BMC Genom..

[17] Tsartsianidou V., Sánchez-Molano E., Kapsona V.V., Basdagianni Z., Chatziplis D., Arsenos G., Triantafyllidis A., Banos G. (2021). A comprehensive genome-wide scan detects genomic regions related to local adaptation and climate resilience in Mediterranean domestic sheep. Genet. Sel. Evol..

[18] Mastrangelo S., Ciani E., Sardina M.T., Sottile G., Pilla F., Portolano B., The Bi. Ov. Ita Consortium (2018). Runs of Homozygosity Reveal Genome-Wide Autozygosity in Italian Sheep Breeds. Anim. Genet..

[19] Mastrangelo S., Tolone M., Sardina M.T., Sottile G., Sutera A.M., Di Gerlando R., Portolano B. (2017). Genome-Wide Scan for Runs of Homozygosity Identifies Potential Candidate Genes Associated with Local Adaptation in Valle del Belice Sheep. Genet. Sel. Evol..

[20] Talebi R., Szmatoła T., Mészáros G., Qanbari S. (2020). Runs of homozygosity in modern chicken revealed by sequence data. G3 Genes Genomes Genet..

[21] Keller M.C., Visscher P.M., Goddard M.E. (2011). Quantification of inbreeding due to distant ancestors and its detection using dense single nucleotide polymorphism data. Genetics.

[22] Nagy I., Nguyen T.A. (2024). Characterizing and Eliminating the Inbreeding Load. Vet. Sci..

[23] Williams J.L., Hall S.J., Del Corvo M., Ballingall K.T., Colli L., Ajmone Marsan P., Biscarini F. (2016). Inbreeding and Purging at the Genomic Level: The Chillingham Cattle Reveal Extensive, Non-Random SNP Heterozygosity. Anim. Genet..

[24] Arias K.D., Fernandez I., Traoré A., Goyache F. (2024). West African Cattle Share Non-Random Heterozygosity-Rich Region Islands Enriched on Adaptation-Related Genes Despite Their Different Origins. Front. Anim. Sci..

[25] Falchi L., Cesarani A., Criscione A., Hidalgo J., Garcia A., Mastrangelo S., Macciotta N.P.P. (2024). Effect of Genotyping Density on the Detection of Runs of Homozygosity and Heterozygosity in Cattle. J. Anim. Sci..

[26] Li G., Tang J., Huang J., Jiang Y., Fan Y., Wang X., Ren J. (2022). Genome-wide estimates of runs of homozygosity, heterozygosity, and genetic load in two Chinese indigenous goat breeds. Front. Genet..

[27] Somenzi E., Senczuk G., Ciampolini R., Cortellari M., Vajana E., Tosser-Klopp G., Pilla F., Ajmone-Marsan P., Crepaldi P., Colli L. (2022). The SNP-Based Profiling of Montecristo Feral Goat Populations Reveals a History of Isolation, Bottlenecks, and the Effects of Management. Genes.

[28] Chessari G., Criscione A., Marletta D., Crepaldi P., Portolano B., Manunza A., Cesarani A., Biscarini F., Mastrangelo S. (2024). Characterization of heterozygosity-rich regions in Italian and worldwide goat breeds. Sci. Rep..

[29] Ruan D., Yang J., Zhuang Z., Ding R., Huang J., Quan J., Gu T., Hong L., Zheng E., Li Z. (2022). Assessment of heterozygosity and genome-wide analysis of heterozygosity regions in two Duroc pig populations. Front. Genet..

[30] Bordonaro S., Chessari G., Mastrangelo S., Senczuk G., Chessa S., Castiglioni B., Tumino S., Marletta D., Criscione A. (2023). Genome-wide population structure, homozygosity, and heterozygosity patterns of Nero Siciliano pig in the framework of Italian and cosmopolitan breeds. Anim. Genet..

[31] Bizarria dos Santos W., Pimenta Schettini G., Fonseca M.G., Pereira G.L., Loyola Chardulo L.A., Rodrigues Machado Neto O., Baldassini W.A., Nunes de Oliveira H., Abdallah Curi R. (2021). Fine-Scale Estimation of Inbreeding Rates, Runs of Homozygosity and Genome-Wide Heterozygosity Levels in the Mangalarga Marchador Horse Breed. J. Anim. Breed. Genet..

[32] Amills M., Capote J., Tosser-Klopp G. (2017). Goat domestication and breeding: A jigsaw of historical, biological and molecular data with missing pieces. Anim. Genet..

[33] EUROSTAT (2023). Goats Population—Annual Data. https://ec.europa.eu/eurostat/databrowser/view/apro_mt_lsgoat/default/table?lang=en.

[34] Gelasakis A.I., Rose G., Giannakou R., Valergakis G.E., Theodoridis A., Fortomaris P., Arsenos G. (2017). Typology and characteristics of dairy goat production systems in Greece. Livest. Sci..

[35] FAO (2022). Domestic Animal Diversity Information System (DAD-IS). https://www.fao.org/dad-is/browse-by-country-and-species/en/.

[36] Krina A., Koutsias N., Pleniou M., Xystrakis F. Climatic classification of Greece: Update—Future estimation—Relation with forest vegetation. Proceedings of the 18th Congress of the Hellenic Forestry Society & International Workshop.

[37] Pappas B.G., Boyazoglu J., Vasiloudis C. (1992). The Skopelos Goat Breed of Greece. Anim. Genet. Resour. Inf..

[38] Nardone A., Ronchi B., Lacetera N., Ranieri M.S., Bernabucci U. (2010). Effects of Climate Changes on Animal Production and Sustainability of Livestock Systems. Livest. Sci..

[39] Cozzi P., Manunza A., Ramirez-Diaz J., Tsartsianidou V., Gkagkavouzis K., Peraza P., Johansson A.M., Arranz J.J., Freire F., Kusza S. (2024). SMARTER-Database: A Tool to Integrate SNP Array Datasets for Sheep and Goat Breeds. Gigabyte.

[40] Chang C.C., Chow C.C., Tellier L.C.A.M., Vattikuti S., Purcell S.M., Lee J.J. (2015). Second-generation PLINK: Rising to the challenge of larger and richer datasets. GigaScience.

[41] Meyermans R., Gorssen W., Buys N., Janssens S. (2020). How to study runs of homozygosity using plink? A guide for analyzing medium density SNP data in livestock and pet species. BMC Genom..

[42] Manichaikul A., Mychaleckyj J.C., Rich S.S., Daly K., Sale M., Chen W.M. (2010). Robust relationship inference in genome-wide association studies. Bioinformatics.

[43] Zhou X., Stephens M. (2012). Genome-wide efficient mixed-model analysis for association studies. Nat. Genet..

[44] Berg P., Groeneveld L.F., Brekke C., Våge D.I., Sørheim K.M., Grøva L. (2020). Genetic characterization of a small closed island population of Norwegian coastal goat. Acta Agric. Scand..

[45] McQuillan R., Leutenegger A.L., Abdel-Rahman R., Franklin C.S., Pericic M., Barac-Lauc L., Smolej-Narancic N., Janicijevic B., Polasek O., Tenesa A. (2008). Runs of Homozygosity in European populations. Am. J. Hum. Genet..

[46] Biscarini F., Manunza A., Cozzi P., Stella A. Common heterozygosity-rich regions across the genomes of commercial and local goat breeds. Proceedings of the 12th World Congress on Genetics Applied to Livestock Production.

[47] Pemberton T.J., Absher D., Feldman M.W., Myers R.M., Rosenberg N.A., Li J.Z. (2012). Genomic patterns of homozygosity in worldwide human populations. Am. J. Hum. Genet..

[48] McLaren W., Gil L., Hunt S.E., Riat H.S., Ritchie G.R.S., Thormann A., Flicek P., Cunningham F. (2016). The Ensembl Variant Effect Predictor. Genome Biol..

[49] Martin F.J., Gall A., Szpak M., Flicek P. (2021). Accessing livestock resources in Ensembl. Front. Genet..

[50] Huang D.W., Sherman B.T., Lempicki R.A. (2009). Bioinformatics enrichment tools: Paths toward the comprehensive functional analysis of large gene lists. Nucleic Acids Res..

[51] Raudvere U., Kolberg L., Kuzmin I., Arak T., Adler P., Peterson H., Vilo J. (2019). G:Profiler: A web server for functional enrichment analysis and conversions of gene lists (2019 update). Nucleic Acids Res..

[52] Wanjala G., Kusuma Astuti P., Bagi Z., Kichamu N., Strausz P., Kusza S. (2023). A review on the potential effects of environmental and economic factors on sheep genetic diversity: Consequences of climate change. Saudi J. Biol. Sci..

[53] Abioja M.O., Logunleko M.O., Majekodunmi B.C., Adekunle E.O., Shittu O.O., Odeyemi A.J., Nwosu E.U., Oke O.E., Iyasere O.S., Abiona J.A. (2023). Roles of candidate genes in the adaptation of goats to heat stress: A review. Small Rumin. Res..

[54] Rangkasenee N., Murani E., Brunner R., Schellander K., Cinar M.U., Scholz A.M., Luther H., Hofer A., Ponsuksili S., Wimmers K. (2013). KRT8, FAF1 and PTH1R gene polymorphisms are associated with leg weakness traits in pigs. Mol. Biol. Rep..

[55] Guang-Xin E., Duan X.H., Zhang J.H., Huang Y.F., Zhao Y.J., Na R.S., Zhao Z.Q., Ma Y.H., Chu M.X., Basang W.D. (2019). Genome-wide selection signatures analysis of litter size in Dazu black goats using single-nucleotide polymorphism. 3 Biotech.

[56] Caroprese M., Ciliberti M.G., Albenzio M., Sevi A. (2017). Climate change impact on immune response in sheep. Sheep Production Adapting to Climate Change.

[57] Naveed M., Kazmi S.K., Amin M., Asif Z., Islam U., Shahid K., Tehreem S. (2018). Comprehensive review on the molecular genetics of autosomal recessive primary microcephaly (MCPH). Genet. Res..

[58] Ding W., Wu Q., Sun L., Pan N.C., Wang X. (2019). Cenpj regulates cilia disassembly and neurogenesis in the developing mouse cortex. J. Neurosci..

[59] Michailidou S., Tsangaris G.T., Tzora A., Skoufos I., Banos G., Argiriou A., Arsenos G. (2019). Analysis of genome-wide DNA arrays reveals the genomic population structure and diversity in autochthonous Greek goat breeds. PLoS ONE.

[60] Colli L., Milanesi M., Talenti A., Bertolini F., Chen M., Crisà A., Daly K.G., Del Corvo M., Guldbrandtsen B., Lenstra J.A. (2018). Genome-wide SNP profiling of worldwide goat populations reveals strong partitioning of diversity and highlights post-domestication migration routes. Genet. Sel. Evol..

[61] Monau P.I., Visser C., Muchadeyi F.C., Okpeku M., Nsoso S.J., Van Marle-Köster E. (2020). Population structure of indigenous southern African goats based on the Illumina Goat50K SNP panel. Trop. Anim. Health Prod..

[62] Browning S.R., Browning B.L. (2012). Identity by descent between distant relatives: Detection and applications. Annu. Rev. Genet..

[63] Bertolini F., Servin B., Talenti A., Rochat E., Kim E.S., Oget C., Palhière I., Crisà A., Catillo G., Steri R. (2018). Signatures of selection and environmental adaptation across the goat genome post-domestication. Genet. Sel. Evol..

[64] Kim E.S., Elbeltagy A.R., Aboul-Naga A.M., Rischkowsky B., Sayre B., Mwacharo J.M., Rothschild M.F. (2016). Multiple genomic signatures of selection in goats and sheep indigenous to a hot arid environment. Heredity.

[65] Mdladla K., Dzomba E.F., Muchadeyi F.C. (2018). Landscape genomics and pathway analysis to understand genetic adaptation of South African indigenous goat populations. Heredity.

[66] Onzima R.B., Upadhyay M.R., Doekes H.P., Brito L.F., Bosse M., Kanis E., Groenen M.A.M., Crooijmans R.P.M.A. (2018). Genome-wide characterization of selection signatures and runs of homozygosity in Ugandan goat breeds. Front. Genet..

[67] Teubner B., Michel V., Pesch J., Lautermann J., Cohen-Salmon M., Söhl G., Jahnke K., Winterhager E., Herberhold C., Hardelin J.P. (2003). Connexin30 (Gjb6)-deficiency causes severe hearing impairment and lack of endocochlear potential. Hum. Mol. Genet..

[68] Lamartine J., Munhoz Essenfelder G., Kibar Z., Lanneluc I., Callouet E., Laoudj D., Lemaître G., Hand C., Hayflick S.J., Zonana J. (2000). Mutations in GJB6 cause hidrotic ectodermal dysplasia. Nat. Genet..

[69] Batissoco A.C., Abreu-Silva R.S., Braga M.C.C., Lezirovitz K., Della-Rosa V., Alfredo T., Otto P.A., Mingroni-Netto R.C. (2009). Prevalence of GJB2 (connexin-26) and GJB6 (connexin-30) mutations in a cohort of 300 Brazilian hearing-impaired individuals: Implications for diagnosis and genetic counseling. Ear Hear..

[70] Gao Y., Wang X., Yan H., Zeng J., Ma S., Niu Y., Zhou G., Jiang Y., Chen Y. (2016). Comparative transcriptome analysis of fetal skin reveals key genes related to hair follicle morphogenesis in cashmere goats. PLoS ONE.

