# Supplementary material for: Genome-Wide Patterns of Homozygosity and Heterozygosity and Candidate Genes in Greek Insular and Mainland Native Goats
Source: Genes (Basel). 2024 Dec 27;16(1):27. doi: 10.3390/genes16010027 (PMC11765163; doi:10.3390/genes16010027)
Supplement: Supplementary file 1 [file genes-16-00027-s001.zip › Suppl Figure S1.pdf]

Supplementary Figure

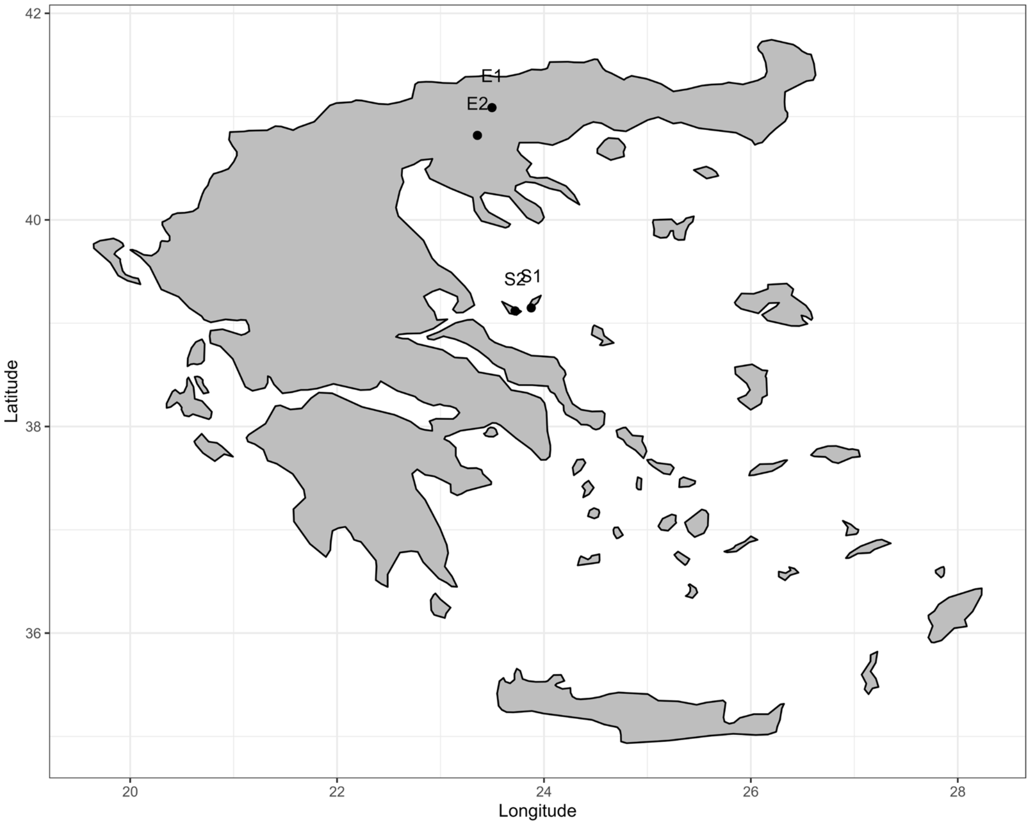

Figure S1. Geographical map indicating sampling locations of Greek goat breeds in the present study. Farms E1 (n= 15) in Serres and E2 in Thessaloniki (n= 55) correspond to the Eghoria breed, while Skopelos goats were sampled from farms S1 (n = 142), S2 (n = 147) located in Alonnisos and Skopelos islands, respectively.
